# Endoscopic Color Doppler Ultrasonographic Evaluation of GastricVarices Secondary to Left-Sided Portal Hypertension

**DOI:** 10.3390/diagnostics4030094

**Published:** 2014-06-26

**Authors:** Takahiro Sato, Katsu Yamazaki, Mutsuumi Kimura, Jouji Toyota, Yoshiyasu Karino

**Affiliations:** Department of Gastroenterology, Sapporo Kosei General Hospital, Kita 3 Higashi 8, Chuo-ku, Sapporo 060-0033, Japan; E-Mails: Katsu.yamazaki@ja-hokkaidoukouseiren.or.jp (K.Y.); kimkimkim66@yahoo.co.jp (M.K.); joji.toyota@ja-hokkaidoukouseiren.or.jp (J.T.); ykarino@ja-hokkaidoukouseiren.or.jp (Y.K.)

**Keywords:** endoscopic ultrasonography, color Doppler, gastric varices, left-sided portal hypertension, splenic vein occlusion

## Abstract

Gastric varices that arise secondary to the splenic vein occlusion can result in gastrointestinal hemorrhaging. Endoscopic color Doppler ultrasonography (ECDUS) was performed in 16 patients with gastric varices secondary to splenic vein occlusion. This study retrospectively evaluated the role of ECDUS in the diagnosis of gastric varices secondary to splenic vein occlusion. Thirteen patients had co-existing pancreatic diseases: 8 with chronic pancreatitis, 4 with cancer of the pancreatic body or tail and 1 with severe acute pancreatitis. Of the remaining 3 patients, 1 had myeloproliferative disease, 1 had advanced gastric cancer, and the third had splenic vein occlusion due to an obscure cause. The endoscopic findings of gastric varices were: variceal form (F) classified as enlarged tortuous (F2) in 12 cases and large, coil-shaped (F3) in 4 cases, and positive for erosion or red color sign of the variceal surface in 4 cases and negative in 12 cases. ECDUS color flow images of gastric variceal flow clearly depicted a round fundal region at the center, with varices expanding to the curvatura ventriculi major of the gastric body in all 16 cases. The velocities of F3 type gastric varices were significantly higher than those of the F2 type. The wall thickness of varices positive for erosion or red color sign was significantly less than the negative cases. I conclude that ECDUS color flow images of gastric variceal flow depicted specific findings of gastric varices secondary to splenic vein occlusion at the round fundal region at the center, with varices expanding to the curvatura ventriculi major of the gastric body.

## 1. Introduction

Gastric variceal hemorrhage is a common complication of portal hypertension and is associated with higher rates of morbidity and mortality than hemorrhage of esophageal varices [1]. Although hemodynamic studies of gastric varices have been made worldwide [2,3,4], splenic vein occlusion is often clinically silent, presenting no obvious symptoms. However, gastric varices secondary to splenic vein occlusion can cause gastrointestinal hemorrhaging (left-sided portal hypertension) [5,6,7,8].

Splenic vein occlusion results in left-sided portal hypertension (characterized by gastric varices, splenomegaly and normal liver function) [9,10,11] that is secondary to various diseases [12,13,14]. The majority of splenic vein occlusions are the result of pancreatic diseases, including acute and chronic pancreatitis and pancreatic tumors.

**Figure 1:**
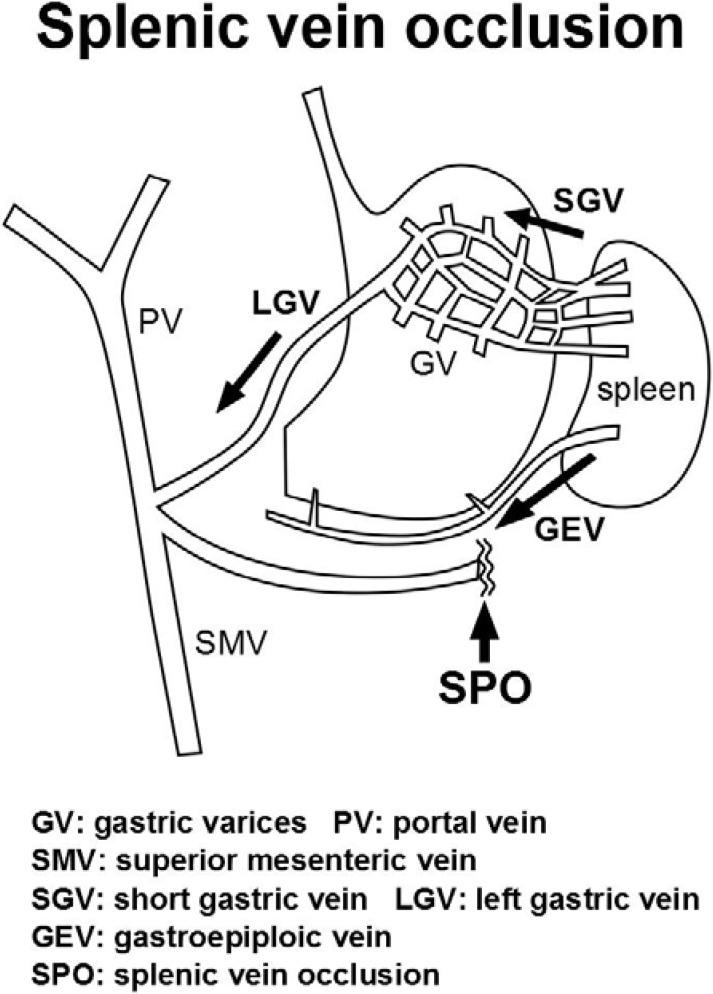
Hemodynamics of gastric varices secondary to splenic vein occlusion (reproduction from Sato *et al.* [16].

Occlusion of the splenic vein causes the splenic venous flow to drain into collateral veins (the short gastric vein and left gastroepiploic vein) and this increased blood flow dilates the submucosal veins of the stomach, resulting in gastric varices (Figure 1). Because blood drainage is diverted by the coronary vein into the patent portal system, the presence of gastric varices without esophageal varices is a very specific sign of splenic vein occlusion [15].

Although splenic vein occlusion is commonly silent clinically, this condition may cause gastrointestinal bleeding from gastric varices. In previous studies of splenic vein occlusion, gastrointestinal bleeding was reported to occur in 16%–78% of patients [8,9,13,17,18,19,20] Sutton *et al.* [9] found 53 cases of such occlusion, and reported a 64% incidence of upper gastrointestinal bleeding. Itzchak and Glickman [20] noted gastrointestinal hemorrhaging in only 3 of 19 patients with splenic vein occlusion. Sarin *et al.* [18] studied the prevalence of gastric varices in 568 patients with portal hypertension and reported that 7 of 9 patients (78%) with gastric varices due to splenic vein occlusion had a history of previous variceal bleeding. With recent improvements in cross-sectional imaging, Heider *et al.* [21] reported gastric variceal bleeding from pancreatitis-induced splenic vein thrombosis (with minimal symptoms) in only 4% of patients. Recently, Butler *et al.* [22] reported that the rate of pancreatitis-induced splenic vein thrombosis associated gastrointestinal bleeding is 12.3%.

The aim of this study was to investigate the role of ECDUS in the diagnosis of gastric varices secondary to splenic vein occlusion.

## 2. Subjects and Methods

### 2.1. Patients

Between January 1996 and December 2013, 205 patients with gastric varices that were found consecutively by routine upper endoscopy were retrospectively evaluated with ECDUS at Sapporo Kosei Hospital. This study group consisted of 16 patients with gastric varices due to splenic vein occlusion (13 males, 3 females, ages 33 to 79 years (mean: 60.5 years)) who were diagnosed by endoscopic color Doppler ultrasono0graphy (ECDUS) and some cases enrolled in my previous study [19] were included in this study. Clinical manifestations and diagnostic evaluation of ECDUS were analyzed retrospectively.

### 2.2. ECDUS

**Figure 2:**
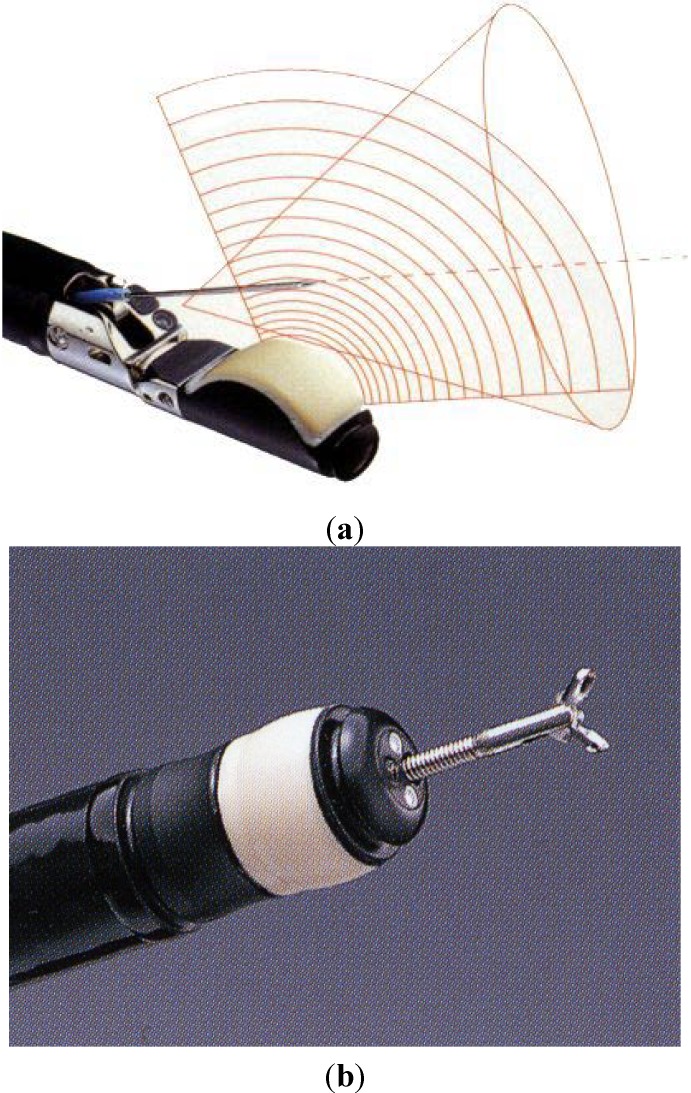
(**a**) PENTAX FG-36UX (forward-oblique viewing), 7.5 MHz, convex type; (**b**) PENTAX EG-3630UR (forward viewing), 10 MHz, electronic radial type.

Hemodynamic evaluation of the gastric varices was performed by ECDUS using a PENTAX FG-36UX (forward-oblique viewing), 7.5 MHz, convex type, which provided 100° images (convex type ECDUS) (Figure 2a) or EG-3630UR (forward viewing), 10 MHz, electronic radial type, which provided 270° images (electronic radial type ECDUS) (Figure 2b) (Pentax Optical, Tokyo, Japan). The HITACHI EUB565 or EUB8500 was used for the display (Hitachi Medical, Tokyo, Japan).

Exploration of gastric varices was conducted by introducing deaerated water from an autoinfuser device through the working channel into the stomach. Evaluation of gastric varices was carried out using ECDUS while the patients remained in a left lateral decubitus position. Initially, identification of gastric varices was made by B-mode scanning followed by color flow mapping. On B-mode scanning, submucosal gastric varices and perigastric collateral veins were obtained as hypoechoic vessels within the gastric wall or in the tissue and spaces exterior to the adventitia of the gastric wall. ECDUS provides a color display of blood flow and evaluates the flow pattern using fast Fourier transform (FFT) analysis. FFT analysis can indicate the flow pattern and calculate the velocity of blood flow. We monitored the color flow images of gastric varices and peri-gastric collateral veins. Velocities were assessed using the pulsed Doppler method, by positioning a sample volume of 1–2 mm in the center of the vessels. The color gain was adjusted so as to eliminate background noise, and the insonation angle was kept below 60° to minimize ambiguity in measurements of blood flow. The mean velocity of blood flow in the gastric varices was obtained by averaging a total of two tracings of conspicuous points (at least three points) on the gastric varices and was selected as the data showing exceedingly high velocity as the blood velocity of patient. The wall thickness of submucosal varices also was evaluated. The wall thickness was obtained at conspicuous points (at least three points), erosion or red color positive site on the gastric varices, and the data were selected showing exceedingly low thickness as the wall thickness of patient.

The absence of a control group of patients with gastric varices due to causes other than splenic vein occlusion is a limitation of this study. The study was performed according to the tenets of the Declaration of Helsinki. Written informed consent was obtained from all patients prior to the procedures. The study was approved by the ethical committee of Sapporo Kosei General Hospital.

## 3. Results

### 3.1. Clinical Details

Among the 16 cases, the reasons for endoscopic examination were as follows: hematemesis in 1 case, tarry stool in 3 cases, screening in 4 cases, and confirmation of gastric varices suspected via computed tomography (CT) in 8 cases. According to ultrasound examinations and CT, or angiographic examination, 13 patients had co-existing pancreatic diseases, including 8 with chronic pancreatitis, 4 with cancer of the pancreatic body or tail and 1 with severe acute pancreatitis. Among the 3 remaining patients, 1 had myeloproliferative disease, 1 had advanced gastric cancer, and the third had splenic vein occlusion due to an obscure cause.

### 3.2. Endoscopic Findings

Endoscopic findings of gastric varices [23] were: variceal form (F) classified as enlarged tortuous (F2) in 12 cases and large, coil-shaped (F3) in 4 cases, and erosion or red color sign of the variceal surface-positive in 4 cases and negative in 12 cases. Three of the 4 cases with erosion or red color sign of the variceal surface had a current history of gastric variceal bleeding.

### 3.3. ECDUS

**Figure 3:**
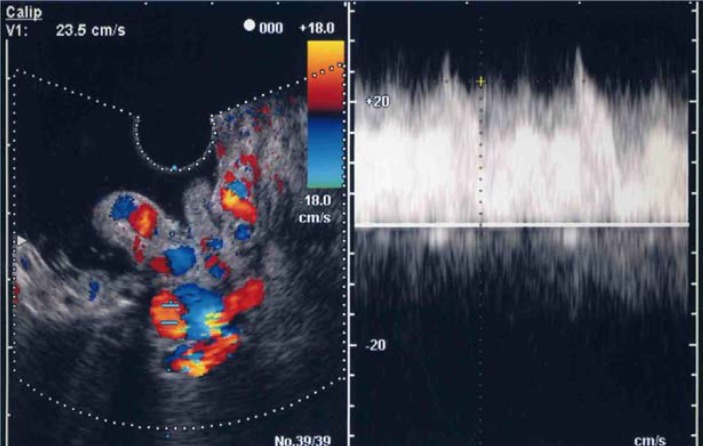
Endoscopic color Doppler ultrasonography showing a color flow image of gastric varices due to splenic vein occlusion that flow as a continuous wave.

**Figure 4:**
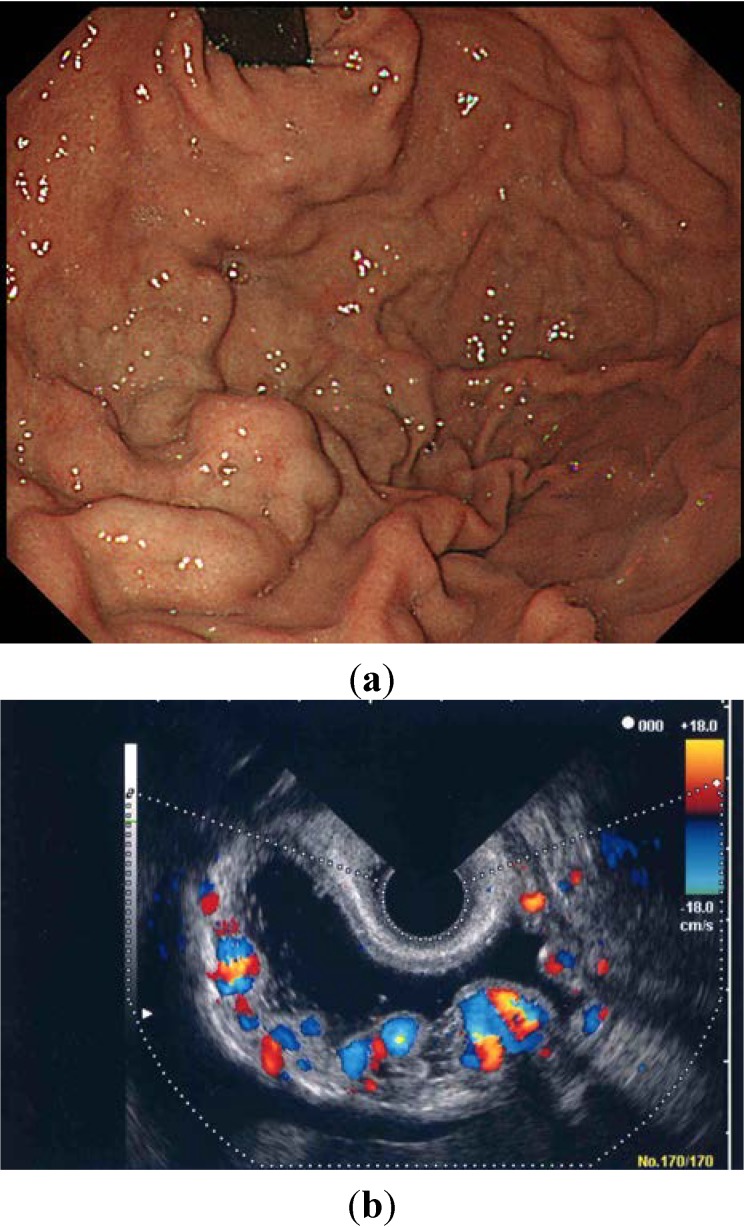
(**a**) Endoscopic findings showing enlarged tortuous, erosion-positive gastric varices in the round fundal region at the center; (**b**) Color flow images from endoscopic color Doppler ultrasonography showing a round fundal region at the center, with varices expanding to the curvatura ventriculi major of the gastric body.

Using ECDUS color flow imaging, gastric varices were delineated in all 16 patients. FFT analysis of variceal blood flow showed a continuous wave in all 16 patients, with flow velocities in the gastric varices ranging between 8.6 and 28.6 cm/s (mean 17.1 ± 4.9 cm/s). Figure 3 shows an electronic radial-type ECDUS image of large, coil-shaped gastric varices located between the fundus and the curvatura ventriculi major of the gastric body, which flows as a continuous wave. Endoscopic findings showed enlarged tortuous, erosion-positive gastric varices in a round fundal region with the pancreatic cancer (Figure 4a). In this case, ECDUS demonstrated clearly gastric variceal color flow images of the round fundal region at the center that expand to the curvatura ventriculi major of the gastric body (Figure 4b). All 16 cases diagnosed as gastric varices secondary to splenic vein occlusion showed similar specific findings on ECDUS color flow images. The ECDUS color flow images of gastric variceal flow depicted specific findings of gastric varices secondary to splenic vein occlusion at the round fundal region at the center, with varices expanding to the curvatura ventriculi major of the gastric body.

We compared the velocities of the gastric varices according to variceal form. The mean velocity of the F3 type gastric varices was 23.0 ± 4.0 cm/s (*n* = 4), while the mean velocity of the F2 type was 15.1 ± 3.4 cm/s (*n* = 12). The velocities of the F3 type were significantly higher than those of the F2 type (*p* < 0.01). Next, we evaluated the wall thickness of submucosal gastric varices. The gastric wall thickness of the submucosal gastric varices was measured at between 0.8 and 2.0 mm (mean 1.5 ± 0.4 mm). The mean wall thickness of red color sign or erosion-positive varices was 1.1 ± 0.2 mm (*n* = 4), while the mean thickness of red color sign or erosion-negative varices was 1.7 ± 0.3 mm (*n* = 12). Thus, the walls of red color sign or erosion-positive varices were significantly thinner than those that were negative (*p* < 0.01).

## 4. Discussion

Gastric varices have been diagnosed by esophago-gastro-duodenoscopy (EGD), a useful modality for observing gastric varices of a certain size and extent. EGD is usually the initial investigation in patients with portal hypertension in order to distinguish between gastric varices and gastric folds, and it has a very sensitive predictive value for variceal hemorrhage [24]. However, cases of red color-positive gastric varices are quite rare and it is difficult to diagnose a high risk of bleeding of gastric varices. Furthermore, EGD is a limited modality for detecting gastric varices, given the depth of the submucosal and extramural collateral veins of gastric varices.

Endoscopic ultrasonography (EUS) has become a useful modality for the diagnosis for esophagogastric varices and is considered most useful for evaluating gastric varices [25,26,27]. By applying EUS with Doppler capabilities, ECDUS allowed the sonographic visualization of the vessels, as well as evaluation of vascular blood flow and morphology. Using B-mode scanning, submucosal gastric varices and perigastric collateral veins were detected as hypoechoic vessels within the gastric wall, or in tissue and spaces exterior to the adventitia of the gastric wall. The gastric variceal channel and the extension of the gastric body can be observed easily with B mode EUS. In a previous report, Sato *et al.* [28] described the utility of ECDUS in patients with gastric varices. Relative to EUS, ECDUS more clearly delineates the visualization of varices, and the color images of blood flow in vessels allow detailed sonographic visualization of the vessels and evaluation of vascular blood flow for the diagnosis of gastric varices.

Endoscopic evidence is not sufficient to distinguish between gastric varices due to splenic vein occlusion or the gastric fold. Additional images resulting from ECDUS color analysis of gastric variceal flow clearly depicted a round fundal region at the center, with varices that expanded to the curvatura ventriculi major of the gastric body on gastric varices due to splenic vein occlusion. The location of ordinary gastric varices was classified as fundal (located far from the cardiac orifice) and cardiac and fundal (located between the cardiac orifice and the fundus) [23], however, there was no case with varices expanding to the curvatura ventriculi major of the gastric body among the ECDUS findings of ordinary gastric varices [28]. These data provide specific findings that may be regarded as hallmarks of gastric varices due to splenic vein occlusion. FFT analysis was used to evaluate the flow pattern and calculate the velocity of blood flow at gastric varices. ECDUS was used to measure the extent and velocity of blood flow in gastric varices, as well as the thickness of the gastric wall to submucosal gastric varices. In addition to a red-color sign obtained during EGD, ECDUS measurements of blood flow velocity and wall thickness in cases with gastric varices may also be useful for determining risks of variceal bleeding, and these findings may be useful in diagnosing the risk of bleeding of gastric varices due to splenic vein occlusion. However, there is no conclusion regarding the relationship between the ECDUS data and bleeding.

In this study, we have expanded the previous case series [19], and the specific ECDUS findings published in the previous paper are similar to those reported here. Also, ECDUS analysis of variceal velocities and wall thickness in this study are similar to the results in another earlier study [28]. It should be noted that the sample size in this report is small and that investigations involving a larger number of patients will be necessary to confirm the results. At present, although there is no consensus on the treatment of gastric varices secondary to splenic vein occlusion, we suggest that this should be targeted at the underlying diseases.

## 5. Conclusions

In conclusion, ECDUS color flow images of gastric variceal flow clearly depicted round fundal regions at the center, with varices expanding to the curvatura ventriculi major of the gastric body as the specific findings of gastric varices secondary to splenic vein occlusion.
